# Phylogeny analysis from gene-order data with massive duplications

**DOI:** 10.1186/s12864-017-4129-0

**Published:** 2017-10-16

**Authors:** Lingxi Zhou, Yu Lin, Bing Feng, Jieyi Zhao, Jijun Tang

**Affiliations:** 1 School of Computer Science and Engineering, Tianjin University, Tianjin, 300072 China; 2 Research School of Computer Science, Australian National University, Canberra, 2601 ACT Australia; 3 University of Texas School of Biomedical Informatics at Houston, Houston, 77030 Texas USA; 4 Department of Computer Science and Engineering, University of South Carolina, Columbia, 29208 South Carolina USA

**Keywords:** Phylogeny reconstruction, Maximum likelihood, Variable length binary encoding, Whole genome duplication

## Abstract

**Background:**

Gene order changes, under rearrangements, insertions, deletions and duplications, have been used as a new type of data source for phylogenetic reconstruction. Because these changes are rare compared to sequence mutations, they allow the inference of phylogeny further back in evolutionary time. There exist many computational methods for the reconstruction of gene-order phylogenies, including widely used maximum parsimonious methods and maximum likelihood methods. However, both methods face challenges in handling large genomes with many duplicated genes, especially in the presence of whole genome duplication.

**Methods:**

In this paper, we present three simple yet powerful methods based on maximum-likelihood (ML) approaches that encode multiplicities of both gene adjacency and gene content information for phylogenetic reconstruction.

**Results:**

Extensive experiments on simulated data sets show that our new method achieves the most accurate phylogenies compared to existing approaches. We also evaluate our method on real whole-genome data from eleven mammals. The package is publicly accessible at http://www.geneorder.org.

**Conclusions:**

Our new encoding schemes successfully incorporate the multiplicity information of gene adjacencies and gene content into an ML framework, and show promising results in reconstruct phylogenies for whole-genome data in the presence of massive duplications.

## Background

Phylogeny analysis is one of the key research areas in evolutionary biology. Currently, the dominant data source used in phylogenetic reconstruction is sequence data [1], which can be collected in large amount at low cost (e.g., for coding genes). However, using sequence data (e.g. gene sequences) in phylogenetic reconstruction needs accurate inference of ortholog relationships and provides us only local information – different parts of the genome may evolve according to different evolutionary models, or even be affected by duplications and losses.

Large-scale changes on genomes may hold the key of building a coherent picture of the past history of contemporary species [2]. In such events, entire segments of a genome may be rearranged, duplicated, or deleted. As whole genomes are collected at increasing rates, whole-genome data has become a new and attractive type of data source for phylogenetic analysis [3–8]. Moreover, researchers uncover links between large-scale genomic events (such as rearrangements, duplications, losses leading to copy number variations) and various diseases, especially cancers. Since phylogenetic reconstruction problem is the key to ancestral reconstruction problem, a number of related works [9–14], based on phylogenetic analysis, have been well studied since the 2010s.

MPBE [5] and MPME [6] introduced the idea of encoding gene orders into aligned sequences without loss of information. Therefore we can use parsimony software such as TNT [15] and PAUP* [8] developed for molecular sequences to reconstruct gene order phylogeny. Although MPBE and MPME failed to compete with direct parsimonious approaches on whole-genome data [3, 4, 16], they show great speedup and pave the way for future improvements. Moreover, sequence data can be analyzed by searching the phylogeny with maximum likelihood score as suggested by Felsenstein [17] in 1981. Recent algorithmic development and high-performance computing tools such as RAxML [18] have made the maximum likelihood approach feasible for analyzing very large collection of molecular sequences and reconstructing better phylogenies than parsimonious methods. The first successful attempt to use maximum-likelihood to reconstruct a phylogeny from the whole-genome data of bacterials was published [19] in 2011, but that method appeared to be too time-consuming to process eukaryotic genomes. Later, Lin et al. [20] described a maximum-likelihood approach, MLWD, for phylogenetic analysis that takes into account genome rearrangements as well as duplications, insertions, and losses. This MLWD approach can handle high-resolution genomes (with tens of thousands of markers) and can be used in the same analysis for genomes with very different numbers of markers [20]. Although the MLWD method outperforms both distance-based methods [21, 22], the MLWD approach did not make full use of the copy number information of both gene adjacency and gene content, and thus its performance fades out when genomes experienced a large number of duplications, especially in the presence of whole genome duplications.

In this paper, we propose new maximum-likelihood methods for phylogenetic reconstruction from whole-genome data, by taking into account copy number variations in both gene adjacency and gene content. Extensive experiments on simulated data sets showed that our new method achieves the most accurate phylogenies compared to existing approaches. Moreover, we also applied our new method to analyze the real whole-genome data from eleven mammals.

## Preliminary

Given a set of *n* genes labeled as *G* = {1, 2,..., *n*}, we represent a genome by an ordered list of these genes, where each gene may appear more than once in a genome. Given a gene *g*, we denote its head by *g* ^*h*^ and its tail by *g* ^*t*^, with *+g* indicating that this gene is oriented from tail to head (from *g* ^*t*^ to *g* ^*h*^) and −*g* indicating otherwise (from *g* ^*h*^ to *g* ^*t*^). An adjacency of two consecutive genes *a* and *b* can form one of the following four possibilities, (*a* ^*t*^,*b* ^*h*^),(*a* ^*h*^,*b* ^*h*^),(*a* ^*t*^,*b* ^*t*^), and (*a* ^*h*^,*b* ^*t*^). A gene *c* lies at one end of a linear chromosome is called a telomere, denoted by a singleton set (*c* ^*t*^) or (*c* ^*h*^). With the above notations, we can represent a genome by a multiset of adjacencies and telomeres (if there’s any). For instance, we represent a simple genome composed of one linear chromosome (+*a*,+*b*,+*a*,−*c*,+*a*) and one circular chromosome (+*d*,−*e*) as a multiset of adjacencies and telomeres *S* = { (*a* ^*t*^), (*a* ^*h*^,*b* ^*t*^), (*b* ^*h*^,*a* ^*t*^), (*a* ^*h*^,*c* ^*h*^), (*c* ^*t*^,*a* ^*t*^), (*a* ^*h*^), (*d* ^*h*^,*e* ^*h*^),(*e* ^*t*^,*d* ^*t*^)}. Note that in the presence of duplicated genes, there is no one-to-one correspondence between genomes and multisets of genes, adjacencies, and telomeres [23]. For example, the genome consisting of the linear chromosome (+*a*,−*c*,+*a*,+*b*,+*a*) and the circular one (+*d*,−*e*), will have the same multiset of adjacencies and telomeres as the above example.

Genome rearrangements change the ordering of genes on a chromosome and exchange or combine content across chromosomes. An inversion or reversal reverses a segment of genes on a chromosome. A transposition swaps two segments on a chromosome. Translocation breaks at two chromosomes and exchange segments between them. An event of fusion concatenates two chromosomes into one, and a fission event is the reverse and splits one chromosome into two.

Deletion, insertion and duplication not only change the ordering of genes, but also change the copy number of genes. A deletion removes one or a segment of genes from a genome, while insertion adds new genes that have not been present into a chromosome at a time. A segmental duplication copies a single or a segment of genes from a genome, and inserted the copy back to the genome. A whole genome duplication (WGD) accounts for the operation on an ancestral node, by which a genome is transformed into another by duplicating all chromosomes.

## Methods

In this section, we first give description of three versions of Variable Length Binary Encoding schemes (VLBE) and then introduce Variable Length Binary Encoding based Phylogeny Reconstruction with Maximum Likelihood on Whole-Genome Data (VLWD*x*).

In the WLMD approach [20], the copy number information of both gene adjacency and gene content has not been fully reflected in the binary encoding. WLMD uses binary encoding to note the absence or presence of an adjacency or gene (i.e., 1 for presence and 0 for absence), but WLMD does not distinguish the number of copies of the same adjacency or gene in the genome.

In this paper, we propose a new encoding scheme that encodes a genome data by Variable Length Binary Encoding schemes (VLBE), which preserves as much as possible of both gene order and gene content information. We then incorporate a dedicated transition model, and develop the phylogenetic reconstruction method, Maximum Likelihood on Whole-Genome Data (VLWD*x*), which is aimed to be more robust compared to WLMD [20], especially in the presence of a large number of duplications.

For rearrangement-only model, we apply *V* *L* *B* *E*_1_ to encode the presence or absence of any adjacency or telomere in the genome. We take into account only the adjacencies and telomeres that appear in at least one of the given genomes. Given *n* distinct genes in all input genomes is *n*, there are *Θ*(*n* ^2^) possible adjacencies and telomeres. However, the number of adjacencies and telomeres that appear in at least one of the input genome is usually much smaller – in fact, it is usually linear in *n* rather than quadratic [20].

For the general model with not only rearrangements, but also duplications, insertions and deletions, we add the encoding of gene content besides the encoding of adjacencies. For each gene, we apply *VLBE*_2_ or *VLBE*_3_ to indicate the presence/absence or the multiplicity of this gene in a genome.

In the following three subsections, we give details on the three encoding schemes, along with the resulting encodings for the genome given in Table 1(a).

**Table 1:** Example of the binary encoding through *V* *L* *B* *E*_1_, for three genomes: *G*_1_: (-2, -1, -3), *G*_2_: (-1, 4, 2), and *G*_3_: (-2, -1, -4, 1, 2)

	Adjacencies							
Encoding	(-3,-2)	(-2,-1)	(-1,-3)	(2,-1)	(-1,4)	(4,2)	(2,-2)	(-1,-4)
*G*_1_	1	01	1	0	0	0	0	0
*G*_2_	0	00	0	1	1	1	0	0
*G*_3_	0	11	0	0	1	0	1	1

Note that (1,2) and (-2,-1) are the same adjacency

### Variable length binary encoding 1 (*VLBE*_1_)

We start with only encoding gene adjacency information. For a dataset *D* of *n* genomes, we scan and collect collect all unique adjacencies to obtain a list *A* of *m* adjacencies. We count the maximum number of occurrences *t* for each adjacency *a*∈*A* among all the genomes. The encoding of each adjacency *a* is performed as follows: if genome *D*_*i*_ has *k* copies of the adjacency *a*, we append *t*−*k* 0’s and *k* 1’s to the sequence.

Table 1 (b) gives an example of *VLBE*_1_ encoding. We can further reduce the length of these sequences by removing those characters at which every genome has the same state and we do this for the next two encoding schemes.

### Variable length binary encoding 2 (*VLBE*_2_)

We propose *VLBE*_2_ to encode the multiplicity of adjacencies as well as the presence or absence of gene content. For an input dataset *D* with *n* genomes, we scan and collect all unique adjacencies to obtain a list *A* of *m* adjacencies. We count the maximum number of occurrences *t* for each adjacency *a*∈*A* among all the genomes. We then perform the encoding of each adjacency *a* as follows: if genome *D*_*i*_ has *k* copies of the adjacency *a*, we append *t*−*k* 0’s and *k* 1’s to the sequence. We also append the encoding of gene content as follows: for each unique gene, if it presents in genome *D*_*i*_, append 1 at the encoding for genome *D*_*i*_, otherwise append 0 to the sequence (see Table 2 for an example).

**Table 2:** Example of the binary sequences using *VLBE*_2_, for three genomes: *G*_1_: (-2, -1, -3), *G*_2_: (-1, 4, 2), and *G*_3_: (-2, -1, -4, 1, 2)

	Adjacencies								Content			
Encoding	(-3,-2)	(-2,-1)	(-1,-3)	(2,-1)	(-1,4)	(4,2)	(2,-2)	(-1,-4)	1	2	3	4
*G*_1_	1	01	1	0	0	0	0	0	1	1	1	0
*G*_2_	0	00	0	1	1	1	0	0	1	1	0	1
*G*_3_	0	11	0	0	1	0	1	1	1	1	0	1

Note that (1,2) and (-2,-1) are the same adjacency

### Variable length binary encoding 3 (*VLBE*_3_)

We further explore whether variable length binary encoding on gene content would also make a difference on phylogeny reconstruction. *VLBE*_3_ is aimed at encoding both adjacencies and gene content. For a dataset *D* with *n* genomes, we scan and collect all unique adjacencies to build a list *A* of *m* adjacencies. We count the maximum number of occurrences *t* for each adjacency *a*∈*A* and encode each adjacency *a* as follows: if genome *D*_*i*_ has *k* copies of adjacency *a*, we append *t*−*k* 0’s and *k* 1’s to the encoding sequence for *D*_*i*_. We also append content encoding in the same way as for the adjacencies. See Table 3 for an example of *VLBE*_3_ encoding.

**Table 3:** Example of binary sequences using *VLBE*_3_, for three genomes: *G*_1_: (-2, -1, -3), *G*_2_: (-1, 4, 2), and *G*_3_: (-2, -1, -4, 1, 2)

	Adjacencies								Content			
Encoding	(-3,-2)	(-2,-1)	(-1,-3)	(2,-1)	(-1,4)	(4,2)	(2,-2)	(-1,-4)	1	2	3	4
*G*_1_	1	01	1	0	0	0	0	0	01	01	1	0
*G*_2_	0	00	0	1	1	1	0	0	01	01	0	1
*G*_3_	0	11	0	0	1	0	1	1	11	11	0	1

Note that (1,2) and (-2,-1) are the same adjacency

## Build phylogeny from sequences

As mentioned above, *VLBE*_1_, *VLBE*_2_ and *VLBE*_3_ aim at transforming gene order information to binary sequences without losing important genomic information, after encoding. The key of phylogenetic reconstruction based on binary encoding is to determine the transition model of flipping a state (from 1 to 0 or from 0 to 1). In order to perform a fair comparison with MLWD, we use the same transition model as described in MLWD [20] here.

Once we build the encoding sequences for all of the input genomes, we use RAxML (version 7.2.8) to reconstruct a tree from these sequences. Although our VLBE encoding may generate a sequence longer than that from other encoding methods mentioned above (up to 2-3 times in all of our experiments), it didn’t significantly increase the running time of RAxML, thanks to RAxML’s excellent implementation on parallel coding.

## Results

### Experiments design

We set to evaluate the performance of our approaches on simulated datasets with known “ground truth”. We further tested our new method on a data set of 11 mammal genomes obtained from Ensembl [24].

We follow the standard practice to set up our simulations [7]. We generate model trees (true trees) with different topologies, then simulate a root genome of *n* genes and perform random evolutionary events (including rearrangements, duplications, insertions and deletions) along each branch to generate child genomes from the root to obtain datasets of leaf genomes. We then reconstruct trees by applying different methods and compare the results against the known evolutionary history.

The simulation process is carried out as follows. First, we produce a birth-death tree *T*, which obeys the same way as described in [20]. Then we find the longest path between two leaf nodes, with length = *K*. We apply different evolutionary rates *r*∈{1,2,3,4} so that the tree diameters are in the range of *d*∈{1*n*,2*n*,3*n*,4*n*}: larger diameter means a genome is more distant from its ancestor, and hence more computationally expensive this data set will be. By timing 1/*K* to tree diameter, we then get the length for a certain branch and we apply a variation coefficient to each branch in this way to vary the length of each branch: for each branch we sample a number *s* uniformly from the interval (−1,1) and multiply the branch length by *e* ^*s*^. Thus, a branch would get its length *L* get by,

$$L = r\times n\times (1/K)\times e^{s} $$

For evolving on each branch, we use a set of evolutionary events, including inversions, fusions, fissions, translocations, indels, segment duplications and whole genome duplications. During the simulation process, each event is assigned a specific value of probability to be selected.

We compare the accuracy of three different approaches, *VLWD*_1_, *VLWD*_2_, *VLWD*_3_ and MLWD. *VLWD*_*x*_ (Variable Length Encoding Whole Genome Data, which corresponds to the encoding schemes *VLBE*_*x*_) is our new approach; MLWD (Maximum Likelihood on Whole-genome Data) is currently the best available method that scales up to analyze thousands of genes and hundreds of leaves.

### Simulation under general model without duplications

We simulate different parameter settings to test our proposed method, and run both our methods and MLWD. Our method outperforms MLWD in every data setting and the improvement is even more significant when the tree diameter gets larger. This result is in line with the observation that variable length binary encoding preserves more adjacency and gene content information than MLWD does.

**Fig. 1:**
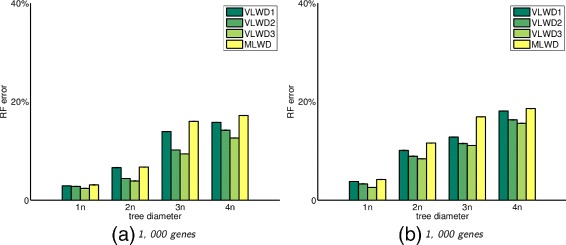
RF error rates for different approaches for trees with 60 species (left) and 100 species (right), with genomes of 1000 genes and tree diameters from 1 to 4 times the number of genes, under the evolutionary events without duplications VLWD1, VLWD2, VLWD3 are the three proposed methods, and MLWD is the previous encoding method

Figure 1 shows error rates for different methods. The x axis indicates the tree diameter and the y axis indicates the RF error rates, which reflects the percentage of different internal edges between two phylogenetic trees [25].

These simulations show that our VLWD approach can reconstruct more accurate phylogenies from genome data experienced various evolutionary events, than the previous binary encoding-based approach MLWD. *VLWD*_3_ also outperforms *VLWD*_1_ and *VLWD*_2_, indicating the importance of encoding the multiplicity of both adjacencies and gene content.

### Simulation under general model with duplications

We generate data sets under a more realistic setting for evolutionary event as well as the genome content. For example, to simulate the evolution of eukaryotic genomes, we generate genome with more than 4,000 genes and the biggest gene family has 20 copies in a single genome.

In our approach, since the encoded sequence of each genome combines information from both gene adjacency and gene content, it is difficult to compute the optimal transition probabilities following the same procedure as described in [20]. Thus we set 1000 as the default bias ratio in the above transition model.

**Fig. 2:**
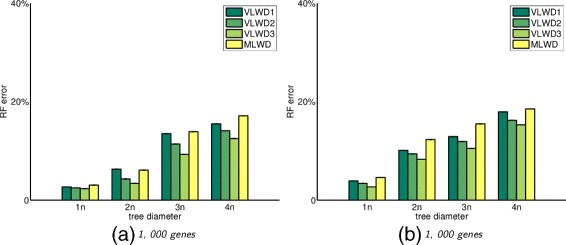
RF error rates for different approaches for trees with 60 species (left) and 100 species (right), with genomes of 1000 genes and tree diameters from 1 to 4 times the number of genes, under the evolutionary events with segmental duplications VLWD1, VLWD2, VLWD3 are the three proposed methods, and MLWD is the previous encoding method

**Fig. 3:**
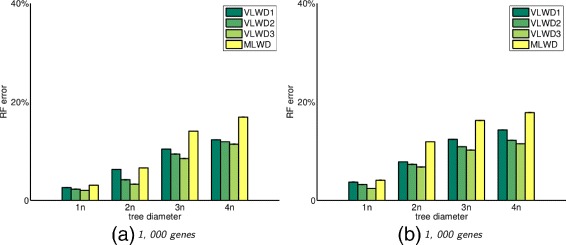
RF error rates for different approaches for trees with 60 species (left) and 100 species (right), with genomes of 1000 genes and tree diameters from 1 to 4 times the number of genes, under the evolutionary events with both segmental and whole genome duplications VLWD1, VLWD2, VLWD3 are the three proposed methods, and MLWD is the previous encoding method

Figures 2 and 3 show the RF error rates. All *VLWD* methods again outperform MLWD, and *VLWD*_3_ always maintains the best performance. Figures 2 and 3 together indicate that MLWD returns similar results for data set with and without whole genome duplication, while *VLWD*_3_ takes advantage of encoding the multiplicity of both gene adjacencies and gene content, and thus improves on the cases with whole genome duplication compared to those without whole genome duplication.

### *VLWD*_3_ phylogeny for eleven mammal genomes

In the previous part, we test our *VLWD*_3_ approach on simulated data set and achieve very good performance for reconstructing phylogenies. Here we test *VLWD*_3_ on the whole genome data of eleven mammal species from online database Ensembl [24].

**Fig. 4:**
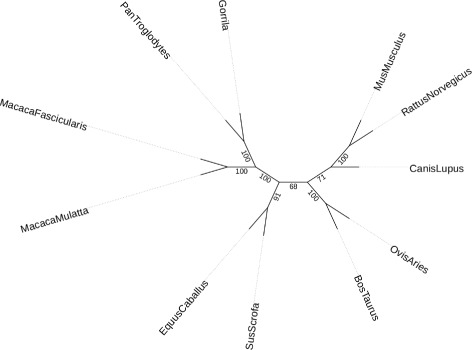
Phylogeny reconstructed by VLWD for eleven mammal genomes, with bootstrap values shown on branches

To obtain the whole genome data of eleven mammal species, we first encode all of the genes into gene orders by using the same gene order to represent all of the homologous genes across different mammal genomes (each genome may contain multiple copies of homologous genes). Subsequently, we input the gene order content and adjacencies into the *VLWD*_3_ approach to reconstruct the phylogenetic relationship for these eleven mammal species (see Fig. 4). Thanks to the efficient implementation of RAxML [18], the running time of *VLWD*_3_ is similar to *MLWD* [20] and *VLWD*_3_ only takes less than ten minutes for the *VLWD*_3_ to output the final solution.

We compare the *VLWD*_3_ phylogeny with the NCBI taxonomy, As Fig. 4 showing, our *VLWD*_3_ approach correctly assign the Macaca mulatta and Macaca fascicularis into the Macaca genus and assign the Pan troglodytes and Gorilla gorilla into the Homininae genus. The Rattus norvegicus and Mus musculus are also been correctly assigned into the subfamily Murinae. The Ovis aries and Bos taurus are also been correctly assigned to the Bovidae family. We also compare this *VLWD*_3_ phylogeny with the previous gene order based mammal phylogeny study of Luo et al. [26]. There are eight mammal species shared by these two phylogenies, and all of the shared branches for these eight species agree with each other. Moreover, two lowest bootstrap scores (68, 71) on the middle two branches in the tree of Fig. 4 reflect the current controversial opinions in placing primates closer to rodents or carnivores [27–32].

## Conclusions

We describe three simple yet powerful approaches for phylogenetic reconstruction based on maximum-likelihood (ML), and design experiments to show the importance of taking into account multiplicities of both gene adjacencies and gene content information. Extensive experiments on simulated data sets show that our proposed approaches achieve the most accurate phylogenies compared to existing methods, particularly in the presence of a large number of duplications or whole genome duplication. Moreover, we applied our new approach to reconstruct the phylogeny of 11 mammal genomes, using only the whole-genome data from Ensembl [24].

Our new encoding schemes successfully model the multiplicities of gene adjacencies and gene content and incorporate them into a maximum-likelihood framework. Experiments on both simulated and real datasets show the effectiveness and efficiency of our approaches in reconstruction phylogenies using whole-genome data, in the presence of massive duplications.
